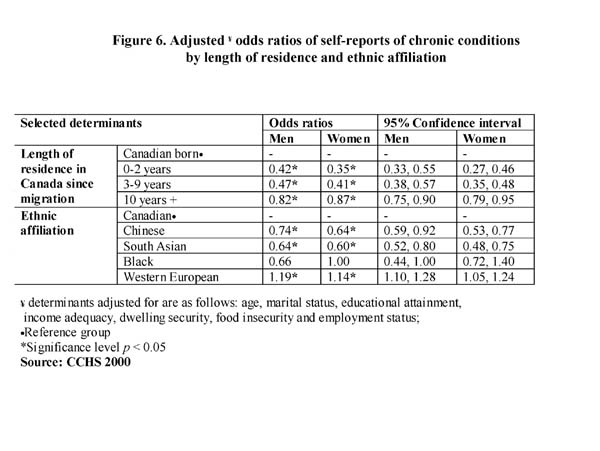# Integrating Ethnicity and Migration As Determinants of Canadian Women's Health

**DOI:** 10.1186/1472-6874-4-S1-S32

**Published:** 2004-08-25

**Authors:** Bilkis Vissandjee, Marie Desmeules, Zheynuan Cao, Shelly Abdool, Arminée Kazanjian

**Affiliations:** 1 School of Nursing Sciences, University of Montreal, Montreal, Canada; 2 Centre for Chronic Disease Prevention and Control, Health Canada, 120 Colonnade Rd, Ottawa, Canada; 3 Centre for Chronic Disease Prevention and Control, Health Canada, 120 Colonnade Rd, Ottawa, Canada; 4 School of Nursing Sciences, University of Montreal, Montreal, Canada; 5 Faculty of Medicine, The University of British Columbia, 5804 Fairview Ave, Vancouver, Canada

## Abstract

**Health Issue:**

This chapter investigates (1) the association between ethnicity and migration, as measured by length of residence in Canada, and two specific self-reported outcomes: (a) self-perceived health and (b) self-reports of chronic conditions; and (2) the extent to which these selected determinants provide an adequate portrait of the differential outcomes on Canadian women's self-perceived health and self-reports of chronic conditions. The 2000 Canadian Community Health Survey was used to assess these associations while controlling for selected determinants such as age, sex, family structure, highest level of education attained and household income.

**Key Findings:**

• Recent immigrant women (2 years or less in Canada) are more likely to report poor health than Canadian-born women (OR = 0.48 CI: 0.30–0.77). Immigrant women who have been in Canada 10 years and over are more likely to report poor health than Canadian-born women (OR = 1.31 CI: 1.18–1.45).

• Although immigrant women are less likely to report chronic conditions than Canadian-born women, this health advantage decreased over time in Canada (OR from 0.35 to 0.87 for 0–2 years to 10 years and above compared with Canadian born women).

**Data Gaps and Recommendations:**

• Migration experience needs to be conceptualized according to the results of past studies and included as a social determinant of health above and beyond ethnicity and culture. It is expected that the upcoming longitudinal survey of immigrants will help enhance surveillance capacity in this area.

• Variables need to be constructed to allow women and men to best identify themselves appropriately according to ethnic identity and number of years in the host country; some of the proposed categories used as a cultural group may simply refer to skin colour without capturing associated elements of culture, ethnicity and life experiences.

## Background

Given the increasing diversity of Canadian society, ethnicity and migration experiences are both important issues to consider when examining the social determinants of women's health. [1-7] Immigrants represent a large and increasing segment of the Canadian population. In 2001, over 5 million Canadians (about 18% of the population) were born outside of the country, and each year approximately 250,000 new immigrants are received. Immigrant women, women refugees and women of diverse ethnic backgrounds form an increasingly large percentage of the Canadian "mosaic": up to 38% of Canadian women are neither French nor British in origin.[8,9] The body of research on the relation between ethnic background and health suggests that immigrant subgroups may be vulnerable in terms of health status, health service use and determinants of health. [7-16] As well, it has been shown that various mechanisms affect the relation between ethnicity, migration and health.[10,11,13]

Health status research suggests that recent (less than 2 years of residence in the host country) immigrants (particularly from non-European countries) are, upon their arrival, healthy overall, with notable exceptions for some health conditions, such as certain infectious diseases. [10-16] However, after 10 years of residence in Canada, the prevalence of a number of chronic conditions and long-term disability approaches the levels found in the Canadian-born population.[12,13] Explanations for this "healthy migrant effect" among recent immigrants may include selection bias and healthy behaviours, such as low rates of tobacco use, as well as spurious research findings due to methodological limitations.[1,2,14,15] However, comprehensive information that includes sex and gender differences in how migrants and ethnic groups experience health is still lacking. More specifically, a number of studies have shown that it is timely to assess the health of women and men with respect to ethnicity and migration experiences, in order to contribute to the understanding of the complexity attached to these concepts.[10,13-15]

Just as gender has been discussed in the various sections of this Report as an essential social determinant of health, diversity is as essential. Indeed, Health Canada, along with the Canadian Centres of Excellence on Migration and the Centres of Excellence for Women's Health Program, strongly recommend a diversity analysis as a complement to the now widely accepted gender-based analysis. [15-17] Incorporating diversity based on ethnicity and migration into health analyses requires accounting for the subtleties of distinction between women and men.[15-19,23]. Diversity results in differential life experiences and opportunities among the heterogeneous group of Canadian women and men. Studies have shown that determinants such as skin colour, immigration and refugee status, country of origin, age at migration and experience with discrimination shape each person's unique identity and, in turn, will affect his or her health and well-being. [22-27] Evidence suggests that migration experiences – above and beyond ethnicity – are as strongly correlated with migrant health as are limited education and lack of access to material resources. While a number of studies have explored ethnicity and health, Cooper and Nazroo indicate that ethnicity has been a neglected dimension in comparative studies of gender and health.[28,29] Similarly, Kinnon, on the basis of her review of the literature, stated that migration experiences have not been systematically investigated in terms of their association with health along with gender and ethnicity.[15,16]

### Immigrants to Canada

In Canada, immigrants are usually "classified" according to four major classes: independent immigrants (including nominated relatives), business class immigrants, family class immigrants (who are sponsored by either independent immigrants or family members who are already residing in Canada) and refugees (who are further subdivided into two classes).[30] China, India, Pakistan, the Philippines and the Republic of Korea were represented among the top 10 source countries for migration to Canada in 2001. The top three regions of origin were Asia and the Pacific (53%), Africa and the Middle East (19%), and Europe and the United Kingdom (17%). In 2001, 61% of immigrants fell into the independent class category, 27% into the family class category, 11% into the refugee category and 1% into a category labelled "other". The leading four source countries for refugees in 2001 were Afghanistan, Sri-Lanka, Pakistan and Yugoslavia.[9,31,32]

The *Immigration Act *establishes a multi-tiered system of rights and privileges among immigrants based on a points system to determine eligibility. Points need to be earned in nine categories, such as education, language and occupation. Usually, when a two-parent family applies for landed immigrant status, only one member in the family is granted the independent status. In most cases, it is the husband who is so designated, because he is perceived to be the head of the household; the wife is categorized as a family class immigrant along with the children. This classification ignores the fact that the wife may have comparable education and work experience to her husband, and may have made an essential contribution to the family income before immigration.[28,33-36]

Migration experiences and their effects on health have received attention very recently.[10,15,29,37] Migrating represents a significant transition in the move from the homeland to a new home, and brings with it many material and psychosocial losses.[22,23,37] This transition can produce profound shifts in people's lives, involving short- to long-term implications for health and well-being. [38-40] Immigrant women may experience increased vulnerability related to settlement, isolation and attainment of the basic needs of the family with limited knowledge of resources in the host country. Such vulnerability potentially exposes them to differing structural, interpersonal, cultural and economic threats to their health and well-being.

Migratory experiences are arbitrarily conceived in three parts: *pre-migratory *includes those experiences that take place from the moment a decision is taken to emigrate (regardless of who in the family unit makes this decision); *settlement experiences *refer to those events after arrival in the host country and up to 5 years after migration; and the *post-migratory experience *refers to years 6 to 10 after migration. Each phase represents different processes of progressive integration and thus should be considered as having differential effects on health.

The label "immigrant woman" does not simply refer to a legal status but encompasses a set of complex realities and experiences. Midiema et al.[41] argue that "immigrant woman" combines technical, legal and social criteria. It is of importance to note that the term "immigrant" often refers to a person who has either acquired permanent resident status in Canada with the rights of Canadian citizens or has actually become a Canadian citizen. On the other hand, when viewed as a social construct, the term "immigrant woman" usually refers to women of colour, women who do not speak English (or French) very well or without an accent other than British or American (or French), and women who occupy lower positions in the occupational hierarchy.[22,23,40]

### The Concept of Ethnicity

There has been much debate surrounding the use of concepts such as "ethnicity", "race" and belongingness to a specific group. Ethnic groups do not refer to homogeneous populations; they include broad categories usually based on age at migration, length of residence, source country/ethnicity, knowledge of host country languages, health status and health behaviour.[10,17,28,36-38] These categories are a useful start in acknowledging diversity among groups of people. Yet these terms also have certain limitations by way of capturing the interaction between biology and the socio-environmental circumstances that are known to contribute to differential experiences in health. [22-24] As Davey Smith rightly points out, recognition of the need to analyze ethnicity and socio-economic position as separate variables in health studies raises a number of issues, including the importance of assessing whether conventional measures of health status and of socio-economic position are sensitive enough for different ethnic groups.[10] Yet another issue is that categories of "race" have often led to the under-counting of those who self-identify outside of the prescribed racial groups (e.g. White, Black, Asian).[39,40] These issues lead to the recognition that different ethnic/racial groups do experience discrimination based on sex and ethnic background. Discrimination can vary in form, depending on how it is expressed, by whom and against whom, and it can occur in all aspects of life. In turn, it affects perceptions of health and health behaviour.[17,22,24,28,35,38-45]

Health is known to be influenced by socio-economic position, and it has also been argued that the socio-cultural context, which includes cultural and migratory experiences, of women and men shapes exposure to health-damaging agents as well as determining individual resources to promote health [17,28,42,46] (see also the chapter on Social Context in this Report). Aside from discrimination, as mentioned above, the literature attests to the importance of cultural variations and migration experience in perceptions of health and what makes one ill or healthy.[17,21,47-53] A number of studies also show that the process of acculturation or progressive integration produces changes in health.[7,54-56] To investigate these changes in health and disease patterns during migration, research has traditionally relied on methods using at least two groups: those born in the host country (a category within which first, second and third generation could be assessed) and those born outside. The number of years spent in a specific country in lieu of assessing acculturation or progressive integration has recently been used to explain determinants/variables of health and well-being of women and men migrating to Canada. Research suggests that different dimensions of the progressive integration process may be associated with different types of outcomes.[17,48,54] However, few studies have investigated health status or chronic conditions as perceived by a diversity of populations and according to the number of years since arrival in a host country.[13,28,29,55-58]

In order to increase our understanding of the social determinants of health in ethnic groups undergoing migration experiences, this chapter seeks to investigate the differential association between self-assessed ethnic affiliation and length of residence in Canada since migration, while controlling for selected socio-economic determinants of health such as age, smoking status and two specific outcomes among Canadian women and men, namely (a) self-perceived health and (b) self-reported chronic conditions.

## Methods

### Data Sources and Measures

Cross-sectional data from the Canadian Community Health Survey (CCHS) – Cycle 1.1 (2000) were analyzed for the purposes of this chapter. The CCHS is a repeated, cross-sectional household-level survey that effectively replaces the cross-sectional component of the National Population Health Survey (NPHS). The sample comprised 125,574 individuals aged 12 years and older at the time of data collection, in 2000–2001. The sample used for the purpose of this chapter included women and men aged 18 to 65 years old and over. Accounting for the complex sample design, weighted methods were used according to CCHS integrated weighting strategies,[59] so that the final results could be generalized to the entire Canadian population.

### Dependent Variables

1. Self-perceived health: respondents assessed their health as excellent, very good, good, fair or poor; in order to assess the profile of the more vulnerable populations, excellent, very good, and good health ratings were combined into one group as well as fair and poor health ratings into one group, which is referred to hereafter as poor-rated health;

2. Self-reported chronic conditions were assessed on a dichotomous scale (yes, no)* (* The list of the reported chronic conditions can be found in Appendix 1).

### Independent Variables

Acknowledging the fact that determinants of health are complex and that variables used for their understanding cannot be compartmentalized into completely independent entities, the categories of sex, gender, ethnicity, migration experience and socio-economic determinants, as listed below, were used.

**Figure 1:**
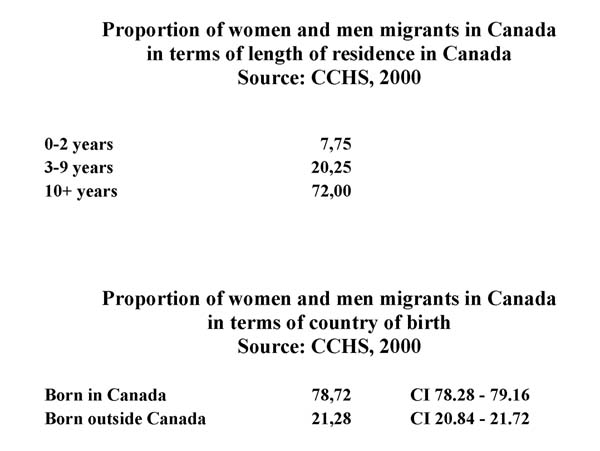


The limited number of women and men who reported being born outside Canada (21.28%, confidence interval [CI]: 20.84, 21.72) as compared with those who reported being born in Canada (78.72%, CI: 78.28, 79.16) (Figure 1) and the need to assess as accurately as possible the contribution of ethnicity and migration experiences as measured by length of residence in Canada, most explanatory variables were reduced to a dichotomous scale for more optimal logistic regression analyses.

1. *Age *– four age groups were selected to reflect different periods across the lifespan and workforce participation: 18–34, 35–44, 45–64, 65+;

2. *Marital status *– dichotomous variable: single; couple;

3. *Educational attainment *– dichotomous variable: less than secondary graduation; some post-secondary school/postsecondary degree;

4. *Income adequacy *– four original categories that were dichotomized: lowest income quartile and lower middle income quartile combined in one category; and upper middle-income quartile and highest income quartile combined in another category;

5. *Food insecurity *– a dichotomous variable asking whether the respondent had had some food insecurity in the previous 12 months;

6. *Dwelling security *– four original categories that were dichotomized: detached house, semi-detached/townhouse and apartment were lumped together to create a category indicating that the respondent did not have any sense of insecurity with respect to his/her dwelling at the time of the survey; the second category referred to the respondent who reported that he/she experienced a sense of insecure dwelling at the time of the survey, namely living in an institution, a mobile home, or some form of collective dwelling;

7. *Employment status *– a dichotomous variable asking whether the respondent worked or not at the time of the survey;

8. *Ethnic affiliation *– a categorical variable asking the respondent to self-identify on the basis of four broad categories: Western European, Chinese, South Asian and Black;

9. *Length of residence *– a categorical variable asking a respondent to choose a category that corresponded to the numbers of years since he/she migrated to Canada (0 to 2 years, 3 to 9 years, and 10 years and more).

### Statistical Analysis

Frequency procedures were used to create tables and calculate the prevalence estimates for each determinant. In accordance with Statistics Canada's guidelines, estimates that were based on a sample of fewer than 30 were deemed unreliable and suppressed. Because socio-economic position has been shown to play a large role in disparities in health, logistic regression models were set up for multivariate analysis to evaluate the effects of covariates on the assessment of self-perceived health and the reported presence of chronic conditions. Confidence intervals for weighted estimates were calculated using the bootstrap method.

## Results

According to the data from CCHS (2000), 20% of women and men reported that they had been in Canada between 3 and 9 years, and 72% of women and men indicated 10 years or more of residence in Canada, leaving 8% of women and men who would be categorized as recent immigrants, that is, 0 to 2 years of residence in Canada since migration (Figure 1).

**Figure 2:**
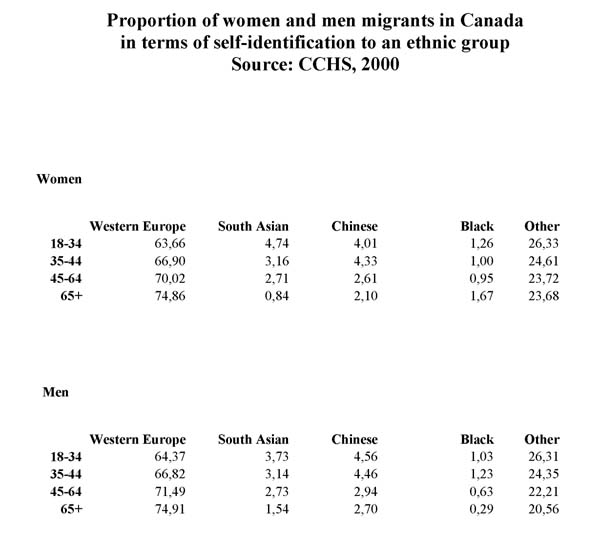


Regarding self-identification to some proposed ethnic categories in the survey, approximately 64% of women and men aged 18 to 34 reported being of Western origin, and that proportion went up to approximately 75% in both groups aged 65 and over (Figure 2). The proportion indicating that they were of Chinese origin ranged from 2% to 5% across the age groups for both sexes. The proportion of South Asian respondents ranged from 1% to 5%. Finally, those who identified themselves as Black were under-represented and made up from 0.3% to 2% of respondents. It is interesting to note that a range of women and men (from 20% to 26%) decided not to choose any of the proposed ethnic affiliation categories.

**Figure 3:**
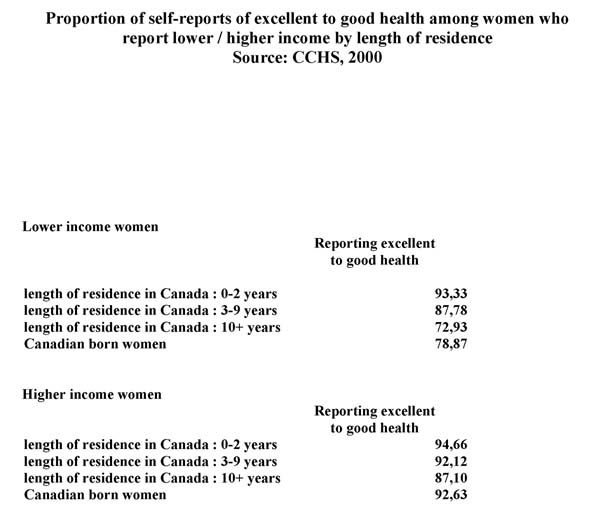


Figure 3 shows that among women born outside Canada and who reported both a lower and a higher income, the proportions reporting good to excellent health decreased with the number of years of residence in Canada; of those reporting lower income, 93% of recent immigrants rated their health as good to excellent, as compared, on the one hand, with 73% of those resident in Canada for 10 years and over and, on the other hand, with 79% of those who reported being Canadian born. Of women with a higher reported income, the proportion of recent immigrants reporting good to excellent health was similar to that of Canadian-born individuals, at 95% and 93% respectively; in the higher income category, 87% of those who reported residing in Canada for 10 years or over rated their health as good to excellent.

**Figure 4:**
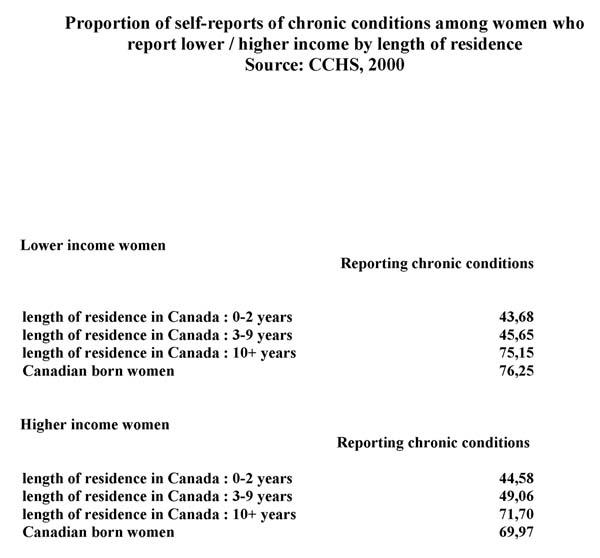


With respect to the reports of chronic conditions, among women who reported both a lower and higher income the proportions with self-reported chronic conditions increased along with the number of years of residence in Canada (Figure 4): of those reporting lower income, 44% of recent immigrants reported the presence of chronic conditions, a proportion that jumped to 75% among women who had lived in Canada for 10 years or longer; this compares with 76% reporting chronic conditions in the Canadian-born group. Of those with higher reported income, 45% of recent immigrants reported the presence of chronic conditions, whereas once again the proportion of women who had lived in Canada for 10 years or more and reported chronic conditions was similar to that of Canadian-born individuals, at 72% and 70% respectively.

**Figure 5:**
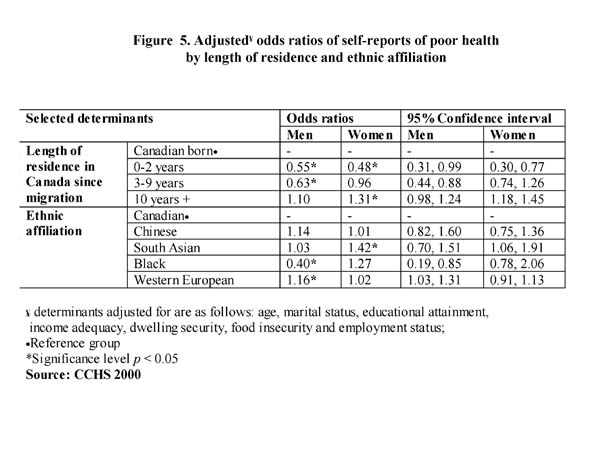


The results of the logistic regression multivariate analyses are presented as odds ratios (OR) and their 95% CIs (Figures 5 and 6). Logistic regression analyses are useful for describing the simultaneous relationship of a group of continuous and/or categorical independent variables and a dichotomous outcome variable, namely self-perceived health and reports of chronic conditions [57,59,61,64,66]. The relative odds express the amount of increase in the outcome that would be produced by one unit increase in the independent variable. In order to avoid obscuring gender and ethnic differences, as can occur when combining both sexes in multivariate models or age-adjusted health outcomes, the models in these analyses were set up separately for women and men.[2,6,7,15,16,27,28] Since a large number of variables were controlled for simultaneously (age and socio-economic position, including employment status, marital status, educational attainment, income adequacy, dwelling security and food insecurity[17,35,38,42,43,46,57,60]), it was deemed important to assess the stability of the model in order to rule out biased results due to multicollinearity. Model fit statistics showed that the models are significant. Pearson correlations were also performed. Coefficients demonstrated weak to moderate associations between each two given variables, indicating a fairly stable multivariate model.

Analyses show that immigrant women who have been in Canada for less than 2 years are less likely to report poor health than Canadian-born women (OR = 0.48, CI: 0.30, 0.77).

As discussed earlier as well as by a number of authors,[10,12-16,21,28,29,37,38,48,51,57-60] this "healthy immigrant effect" disappears over time. Women who have migrated to and resided in Canada for at least 10 years and over are more likely to report poor health than Canadian-born women (OR = 1.31, CI: 1.18,1.45). Among men, the protective effect of length of residence wears out from between 2 and 9 years of residence, but no significant differences are shown after 10 years and over of residence in Canada between the long-term migrant men and Canadian-born men.

With respect to the association of ethnic affiliation and self-perceived health, controlling for confounders, women who indicated that they were of South Asian origin were more likely than Canadian women to report poor health (OR = 1.42, CI: 1.06,1.91). Among men, those who identified themselves as Black were less likely than their Canadian counterparts to report poor health, whereas the opposite was observed for men of Western European origin (OR = 1.16, CI: 1.03,1.31).

Controlling for confounders as indicated above, clearer patterns by length of residence in Canada were observed for both women and men when self-reported chronic conditions were considered as an outcome variable. Women and men were both less likely than Canadian-born individuals to report chronic conditions. However, as the years of residence in Canada increase, the protective effect becomes progressively less for both groups. This gradient shows a gradual loss over time of the health advantage that migrant women (for 0–2 years of residence, OR = 0.35, CI:0.27, 0.46 and for 10+ years of residence, OR = 0.87, CI:0.79, 0.95) and men (for 0–2 years of residence, OR = 0.42, CI:0.33,0.55 and for 10+ years of residence, OR = 0.82, CI:0.75, 0.90) are known to enjoy upon arrival in the host country.

With respect to the association between ethnic affiliation and self-report of chronic conditions, controlling for confounders, women who indicated that they were of South Asian (OR = 0.60, CI: 0.48,0.75) as well as of Chinese (OR = 0.64, CI: 0.53,0.77) origin were less likely than Canadian women to report chronic conditions. On the other hand, women self-assessing themselves as Western European were more likely to report chronic conditions (OR = 1.14, CI: 1.05,1.24). Interestingly, a similar pattern between the same ethnic affiliation groups and Canadian men was observed.

## Discussion

One limitation of our analysis is that the use of cross-sectional data makes it difficult to disentangle the direction of causality and thus limits the ability to exclude the potential for reverse causation.[8,10-13] Nonetheless, controlling for determinants such as age and socio-economic position, complex variables such as ethnic affiliation and/or country of origin remain associated with self-reported measures of health known to be rather subjective and culturally bound measures of health.[13,17,28,29,42,43,61] Women who had been in Canada for at least 10 years and over were significantly more likely to report poor health than recent immigrant women; for men, being in Canada at least 10 years and over did not make a significant difference. This attests to the differential needs and patterns of reporting unmet needs of care between women and men who experience migration.

These results are convergent with the study by Dunn and Dyck[13] of the social determinants of health in Canada's immigrant population using the NPHS (1994–1995). They found some consistent pattern for length of residence in Canada and reports of chronic conditions by women and men. Their data also showed that as length of residence increased, women and men were more likely than the Canadian-born population to report poor health status. They explain these findings by the fact that age is certainly associated with reports of poorer health status among the migrant population, as is the case with older Canadians in general.

With respect to self-reports of chronic conditions, women who had experienced migration were less likely to report chronic conditions than Canadian-born women, and the gradient among women according to their years of residence in Canada was steeper than for men. Furthermore, women and men identifying themselves as having Western European origin were more likely to report chronic conditions. Similar results were obtained by Dunn and Dyck using the NPHS.[13] Our descriptive data showed that those who identified themselves as having Western European origin were older and more established immigrants, and this may partly explain the likelihood of reports of chronic conditions among women and men who migrated to Canada a long time ago.

These trends in research results raise the possibility that socio-economic inequalities cannot fully explain ethnic inequalities in health.[51,54] This very measure is culturally bound. As Nazroo points out,[51] "It is important to recognize that the process of standardization for socio-economic position when making comparisons across groups, particularly ethnic groups, may not be so straightforward." In cross-cultural research, the heterogeneity of women and men who constitute the population experiencing migration needs to be accounted for, not only with respect to their country of birth, but also the country of origin, the migratory trajectory and the actual experiences of migration as they relate to health.

Variables such as migration, as measured here by length of residence in Canada, as well as self-reports of ethnic affiliation are indeed complex variables; they need to be examined in conjunction with variables such as age at migration, conditions of migration and socio-economic conditions across the life course.[22,25,27] The progressive integration process also needs to be examined with a more systematic gender perspective. Language spoken inside and outside the home, values and religious observances may be associated with different aspects of the progressive integration process; the latter may occur differentially between women and men. Indeed, the relation between taking on host country culture and relinquishing the culture of origin is not reciprocal, as a strict assimilation model would imply; therefore, acculturation is not a linear process, simultaneously invoking health risk and protective environment.[14,17,61-67] Furthermore, it has also been shown that ethnic differences in self-reported health cannot be assumed to be a consequence of cultural differences between immigrant groups.[22-24,28,38,54,61]

Examining migration as a determinant of health requires more research and debate on the methods of measuring ethnicity and migration variables that would better account for gender and diversity in the experiences of migration as time of residence in a host country increases. Some have argued that it is time to abandon the assessment of race/ethnicity in public health research.[68] Others, including a number of authors in this Report, insist that collecting gender, ethnicity and migration data along with other social determinants of health is necessary to the creation of gender sensitive and culturally appropriate interventions of public health surveillance that contribute to the elimination of ethnic inequalities in health.[14,17,21-23,29,42,43,47,51,55]

### Data Gaps and Recommendations

• Current surveillance methods need to continue and include representative samples of subgroups in the Canadian population; the heterogeneity of women and men experiencing migration requires that large enough samples of women and men from different countries of birth as well as countries of origin are selected so that culturally sensitive conclusions can be drawn and allow for a proper analysis of the health determinants of women from diverse ethnic backgrounds and/or experiences of migration.

• Migration experience needs to be conceptualized according to the results of past studies and included as a social determinant of health above and beyond ethnicity and culture. It is expected that the upcoming longitudinal survey of immigrants will help enhance surveillance capacity in this area.

• In order to account for the cultural differences in the pathways of experiencing health and chronic conditions and because these experiences are relational and illustrate the underlying mechanisms for the differential distribution of health by socio-economic position, it is important to adopt not only a gender sensitive approach to the development of indicators but also a broader diversity interpretation of the health effects of potential advantageous and disadvantageous conditions – for example, to try and account for the social capital of women and men who undergo experiences of migration (network, resilience, ease of access, differential degree of control of resources by women, men and other members of the family).

• Issues such as accessibility (cultural, geographic, linguistic, financial), appropriateness and adequacy of health services are unlikely to be unique to women and men experiencing migration and need to be systematically accounted for.

• There should be consideration of the underlying socio-economic and socio-political forces that shape the life conditions of women and men who are from a diverse background and/or undergo migration.

• Variables need to be constructed to allow women and men to best identify themselves appropriately according to ethnic identity and number of years in the host country; some of the proposed categories used as a cultural group may simply refer to skin colour without capturing associated elements of culture, ethnicity and life experiences.

• Variables need to be constructed to reflect the ability to cope with a physically or psychologically hazardous work environment, which has been shown to be associated with a better trajectory of healthy life in a new country and/or culture.

• Research designs need to account for the fact that reporting of perceived health and presence of chronic conditions is sensitive to gender, culture and ethnicity as well as migration experience.

• A variety of research strategies are needed that should include a diversity of women and men to collaborate in the development, design, and implementation of national monitoring.

## Figures and Tables

**Figure 6:**